# Perioperative advanced haemodynamic monitoring of patients undergoing multivisceral debulking surgery: an observational pilot study

**DOI:** 10.1186/s40635-023-00543-1

**Published:** 2023-09-08

**Authors:** Charlotte Middel, Matthias Stetzuhn, Nadine Sander, Björn Kalkbrenner, Timo Tigges, Alexandru-Gabriel Pielmus, Claudia Spies, Klaus Pietzner, Michael Klum, Clarissa von Haefen, Oliver Hunsicker, Jalid Sehouli, Frank Konietschke, Aarne Feldheiser

**Affiliations:** 1 Department of Anaesthesiology and Operative Intensive Care Medicine (CCM, CVK), Charité, Universitätsmedizin Berlin, corporate member of Freie Universität Berlin, Humboldt-Universität Zu Berlin, and Berlin Institute of Health, Berlin, Germany; 2 Department of Electronics and Medical Signal Processing, Technical University, Berlin, Germany; 3 Department of Gynaecology With Center for Oncological Surgery, Campus Virchow Klinikum, Charité, Universitätsmedizin Berlin, Berlin, Germany; 4 Institute of Biometry and Clinical Epidemiology, Charité, Universitätsmedizin Berlin, corporate member of Freie Universität Berlin, Humboldt-Universität Zu Berlin, and Berlin Institute of Health, Berlin, Germany; 5 Department of Anaesthesiology, Intensive Care Medicine and Pain Therapy, Evangelische Kliniken Essen-Mitte, Huyssens-Stiftung/Knappschaft, 45136 Essen, Germany

**Keywords:** Perioperative medicine, Electrical cardiometry, Multivisceral cancer surgery, Ovarian cancer, Haemodynamic monitoring, Perioperative haemodynamics, Postoperative complications

## Abstract

**Background:**

Patients undergoing high-risk surgery show haemodynamic instability and an increased risk of morbidity. However, most of the available data concentrate on the intraoperative period. This study aims to characterise patients with advanced haemodynamic monitoring throughout the whole perioperative period using electrical cardiometry.

**Methods:**

In a prospective, observational, monocentric pilot study, electrical cardiometry measurements were obtained using an Osypka ICON™ monitor before surgery, during surgery, and repeatedly throughout the hospital stay for 30 patients with primary ovarian cancer undergoing multivisceral cytoreductive surgery. Severe postoperative complications according to the Clavien–Dindo classification were used as a grouping criterion.

**Results:**

The relative change from the baseline to the first intraoperative timepoint showed a reduced heart rate (HR, median – 19 [25-quartile − 26%; 75-quartile − 10%]%, *p* < 0.0001), stroke volume index (SVI, − 9.5 [− 15.3; 3.2]%, *p* = 0.0038), cardiac index (CI, − 24.5 [− 32; − 13]%, *p* < 0.0001) and index of contractility (− 17.5 [− 35.3; − 0.8]%, *p* < 0.0001). Throughout the perioperative course, patients had intraoperatively a reduced HR and CI (both *p* < 0.0001) and postoperatively an increased HR (*p* < 0.0001) and CI (*p* = 0.016), whereas SVI was unchanged. Thoracic fluid volume increased continuously versus preoperative values and did not normalise up to the day of discharge. Patients having postoperative complications showed a lower index of contractility (*p* = 0.0435) and a higher systolic time ratio (*p* = 0.0008) over the perioperative course in comparison to patients without complications, whereas the CI (*p* = 0.3337) was comparable between groups. One patient had to be excluded from data analysis for not receiving the planned surgery.

**Conclusions:**

Substantial decreases in HR, SVI, CI, and index of contractility occurred from the day before surgery to the first intraoperative timepoint. HR and CI were altered throughout the perioperative course. Patients with postoperative complications differed from patients without complications in the markers of cardiac function, a lower index of contractility and a lower SVI. The analyses of trends over the whole perioperative time course by using non-invasive technologies like EC seem to be useful to identify patients with altered haemodynamic parameters and therefore at an increased risk for postoperative complications after major surgery.

**Supplementary Information:**

The online version contains supplementary material available at 10.1186/s40635-023-00543-1.

## Background

Mortality after surgery is still high in Europe [1] and haemodynamic monitoring was shown to reduce postoperative mortality in high-risk surgical patients and procedures [2]. A study showed reduced postoperative complications through a haemodynamic algorithm based on non-invasively measured preoperative values [3]. It indicates that the consideration of advanced haemodynamic values throughout the whole surgical pathway, from preoperative to postoperative values, holds a promising approach.

Cytoreductive surgery for ovarian cancer is often extensive in scale, leading to significant fluid shift or loss. Thus, it is associated with a high risk of postoperative complications. A rate of complications ranging from 9.5 to 47%, or more specifically of severe complications from 5.5 to 19%, are reported [4–6]. Most patients undergoing cytoreductive surgery due to ovarian carcinoma do not have major cardiovascular disease [4], meaning they can compensate for changes, such as significant blood loss, for an extended time, before heart rate and blood pressure decompensate.

Electrical cardiometry (EC) is an algorithm for non-invasive and continuous cardiac output (CO) monitoring based on thoracic bioimpedance. A recent meta-analysis showed a low bias of CO measurements between measurements of EC, intermittent pulmonary artery, and transpulmonary thermodilution, but wide limits of agreements and a high mean percentage error [7]. Yet, it should also be considered, that this calculated error rate is comparable to error rates of other minimal or non-invasive monitoring techniques that are already established in clinical use [7, 8]. Furthermore, most studies only evaluate the absolute CO value and do not sufficiently evaluate trends or the value of other parameters. While it may not replace transpulmonary thermodilution for the measurement of absolute CO values, it might still be applicable as a trend monitor measuring acute changes in CO, which is relevant for clinical decision-making [7, 9, 10]. As a non-invasive methodology, EC is easily applicable and carries little to no risk for the patient. Our group, therefore, used EC in an exploratory fashion during the perioperative course of patients undergoing cytoreductive surgery for ovarian cancer to examine advanced haemodynamic parameters.

We hypothesised that advanced cardiac monitoring using EC unveils haemodynamic alterations from the preoperative to the intraoperative stage as well as changes during the postoperative phase that are so far not described in the literature. We also aimed to explore differences in clinical, haemodynamic, and immunological features in patients with and without postoperative complications, hoping to improve the detection of complications and evaluating the feasibility of preoperative monitoring to reduce complications in the future.

## Materials and methods

### Study design and participants

This was a prospective, observational, monocentric pilot study at the Campus Virchow-Klinikum of the Charité—Universitätsmedizin Berlin, Germany. Ethical approval for this study was provided by the responsible ethics committee of the Charité—Universitätsmedizin Berlin, Germany (Ethics committee N° EA1/390/16, Chairman Prof. Dr. med R. Uebelhack) on December 14, 2016. The study was registered internationally prior to the start of the trial (ClinicalTrials.gov ID: NCT03131102, principal investigator: Aarne Feldheiser, date of registration: March 27, 2017) and was carried out following the principles of the Declaration of Helsinki. The trial was performed from August 29, 2017, to October 09, 2018, at the Campus Virchow-Klinikum, Charité—Universitätsmedizin Berlin, Germany. Eligible patients were adults receiving debulking surgery and subsequent chemotherapy due to primary ovarian cancer, who gave their informed written consent to study participation. Not eligible were patients with recurrent ovarian cancer or when postoperative chemotherapy was not intended. Further criteria for exclusion were an ASA-Score ≥ 4, chronic heart failure NYHA IV, radiological evidence of pulmonary oedema, dialysis-dependent kidney insufficiency, atrial fibrillation, diabetic neuropathy, intolerance to colloidal fluids, neurological or psychiatric disorders limiting their legal capacity, being institutionalised due to official order, or being an employee of the Charité. The medical reasons for exclusions mentioned above stem from the possible interference of significant cardiovascular disease or cardiac arrhythmias with high-quality data analysis using EC monitoring. There was no financial compensation for enrolling in the study. As this was an observational study, all patients received the standard of care concerning surgery and medical care. The patients were evaluated, prepared for the operation, and treated according to the published and certified standards (according to DIN EN ISO 9001) of the Department of Anaesthesiology and Intensive Care Medicine, Campus Virchow-Klinikum and Campus Charité Mitte, and of the Department of Gynaecology with Center for Oncological Surgery, Campus Virchow-Klinikum, Universitätsmedizin Berlin, Germany. The treating physicians were blinded to the data collected and no diagnostic or treatment decisions were made based on our data.

### Data collection

Patients were screened and included in the study the day before surgery. EC measurements were performed with an Osypka ICON™ monitor (Osypka Medical GmbH, Berlin, Germany, Software Version 3.11.1) for 20 min on the day before surgery (Baseline = BL), 1 h (Postanaesthesia care unit timepoint 1 = PACU T1) and 4 h (PACU T2) after surgery, and on the postoperative days 1, 2, 3 and 7 (POD1-3, 7). During surgery (OP) and the first night postoperatively (N), EC measurements were performed continuously and later split into parts of 30 min (OP1-6) or 6 h (N1-2), respectively. The intraoperative measurement was started after the induction of anaesthesia. Fluid and drug administrations of any kind were recorded. In addition, the Sequential Organ Failure (SOFA) and the Acute Physiology and Chronic Health Evaluation II Score (APACHE II Score) were evaluated to assess the statistical risk of mortality. Blood samples were drawn at OP, T1, POD1, and POD3 and analysed regarding inflammatory markers. In the end, data from surgery reports, discharge summaries, and tumour conferences were collected and postoperative complications were classified according to Dindo et al. [11]. Because of the extent of surgery performed, only complications of Clavien–Dindo IIIa or higher were considered relevant. The occurrence of complications was subsequently taken as a binary grouping criterion. Finally, all data collected were validated by the study team and digitised.

### Electrical cardiometry

Electrical cardiometry is an algorithm for estimating stroke volume and cardiac output from non-invasive and continuous measurements of thoracic bioimpedance. Several devices measuring EC are commercially available, we used the Osypka ICON™ monitor. The method of EC was thoroughly explained previously [12]. In short, setting up the measurement requires little training and is possible within a few minutes, as only four adhesive single-use electrodes need to be applied to the patient. Through these electrodes placed on the neck and thorax, an alternating current is applied to the patient and the resulting voltage is measured. Their ratio gives the electrical impedance. Changes in impedance in relation to the cardiac cycle are recorded over time in a continuous fashion and used to calculate various haemodynamic parameters [12]. A description of the relevant parameters can be found in the supplement (Additional file 1: Text S1 [13–22] and Additional file 2: Table S1 [23, 24]).

### Statistical analysis

Data processing and analysis were performed using the programming language R [25] for statistical computing (R-packages used were Gmisc, Hmisc, nparLD [26], coin, tableone, htmlTable, tidyverse, ggpubr, HRM [27], fs, shiny, knitr, rmarkdown, rstatix, scales, gridExtra) and the software R Studio^®^ [28].

For the analysis of the main topic of the study, a non-parametric longitudinal data analysis in a two-factorial experiment was used to assess changes in the EC parameters over time (dependent factor time) and additionally, differences between groups (independent factor group), and their interactions [26]. Also, this test was used for direct and relative comparisons of the EC parameters to the preoperative baseline and the analysis of IL-6 and ICAM-1. Due to the low number of data points, the longitudinal data were also analysed by the R package HRM [27], which is also suitable to analyse non-normally distributed data using non-parametric testing. The results showed no relevant differences between the methods used. Thus, only the calculations of the nparLD [26] package are presented.

Differences for secondary endpoints between patient groups were evaluated using the Wilcoxon–Mann–Whitney rank-sum test for metric data and the Fisher’s exact test for categorical variables.

A two-tailed *p*-value of less than 0.05 was considered statistically significant. Still, due to the study design, all *p*-values feature an exploratory character and thus, do not allow for generalisation or proof. For the same reason, no alpha-adjusting for multiple testing was conducted. The data are expressed as median [quartiles] or frequency (%) according to their scaling.

## Results

Thirty patients were initially enrolled in the study. One patient was excluded from the analysis, due to her cancer being too far advanced in surgical staging for the debulking surgery to be conducted. Overall, data from 29 patients were included in the analysis. Due to technical errors during EC recordings, there are only data from 28 (96.5%) patients during surgery. For postoperative timepoints 26 of the 29 (89.6%) measurements were acquired on average. Seven patients (24.1%) suffered from postoperative complications classified according to the Clavien–Dindo classification of IIIa or higher and formed the group of patients with complications. During the hospital stay, one patient (3.4%) died of a postoperative complication due to multiorgan failure, while no subject in the group without complications died (*p* = 0.241).

**Fig. 1:**
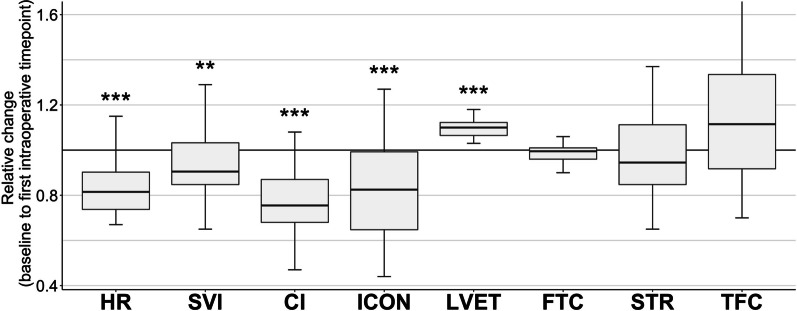
Relative change from the baseline to the first intraoperative timepoint (OP1). At that intraoperative timepoint, the patients received a continuous norepinephrine administration rate of median 0 [Q25 0; Q75 0.02] µg kg^−1^ min^−1^. Data are shown as boxplots. Asterisks indicate (**) *p* < 0.01 and (***) *p* < 0.001 of the first intraoperative versus preoperative baseline values according to the non-parametric analysis for longitudinal data. *CI* cardiac index, *FTC* corrected flow time, *HR* heart rate, *ICON* index of contractility, *STR* systolic time ratio, *SVI* stroke volume index, *TFC* thoracic fluid content

The relative change from the baseline to the first intraoperative timepoint (Fig. 1) showed a reduced heart rate (HR, median − 19% [Q25 − 26%; Q75 − 10%], *p* < 0.0001), stroke volume index (SVI, − 9.5 [− 15.3; 3.2] %, p = 0.0038), cardiac index (CI, − 24.5 [− 32; − 13] %, *p* < 0.0001) and index of contractility (− 17.5 [− 35.3; − 0.8] %, *p* < 0.0001). The infusion rate of norepinephrine during the first thirty minutes intraoperatively amounted to a median of 0 [0 to 0.02] µg kg^−1^ min^−1^. A prolonged left ventricular ejection time is to be expected when the HR is diminished and was further underpinned by the unchanged flow time corrected for HR.

**Fig. 2:**
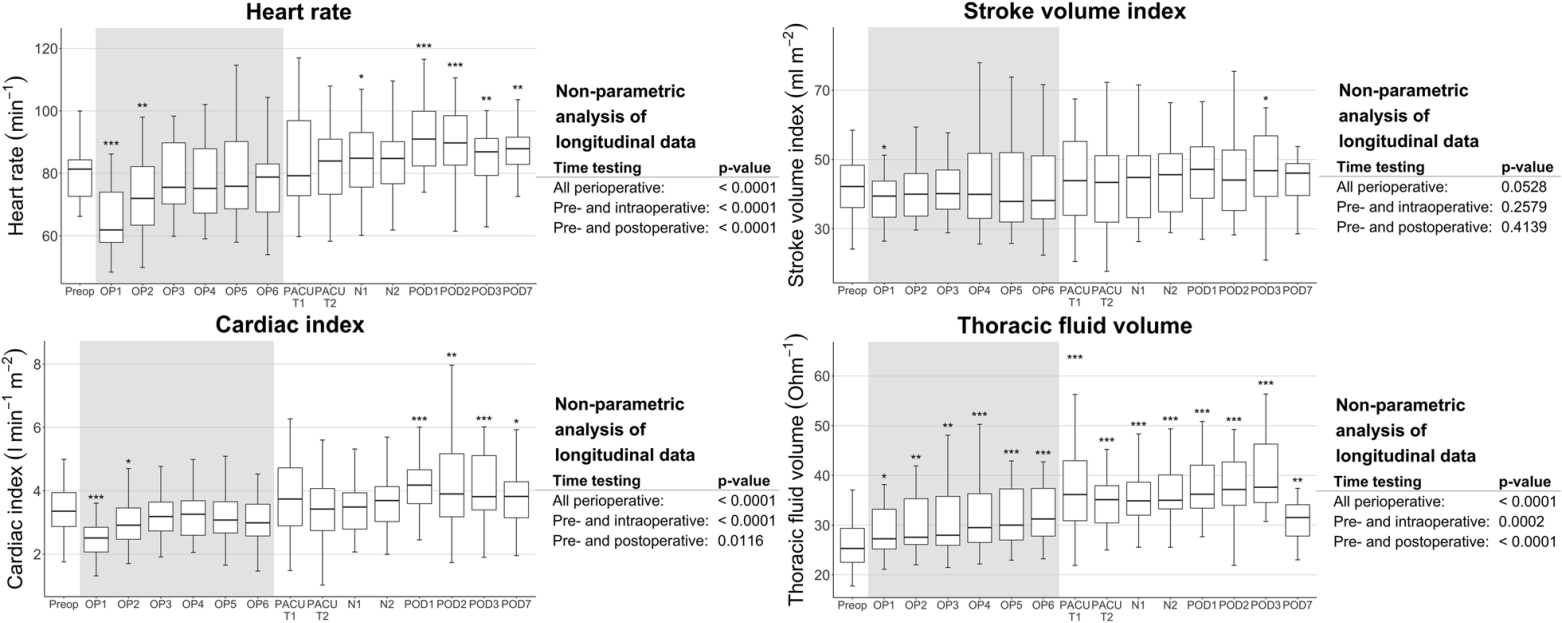
Perioperative time course of selected EC parameters (heart rate, stroke volume index, cardiac index, thoracic fluid content). Data are shown as boxplots over time from the preoperative baseline up to the postoperative day 7 (POD7). Asterisks indicate (*) *p* < 0.05, (**) *p* < 0.01, and (***) *p* < 0.001 of the respective timepoint versus the preoperative baseline values according to the non-parametric analysis for longitudinal data. The non-parametric analyses of longitudinal data for the parameters over the perioperative period are indicated on the right-hand side, respectively. *BL* baseline, *N1-2* timepoints 1–2 during the first postoperative night, *OP1-6* timepoints 1–6 in surgery, *PACU T1* postanaesthesia care unit timepoint 1, *PACU T2* postanaesthesia care unit timepoint 2, *POD1-3/7* postoperative days 1–3/7

**Fig. 3:**
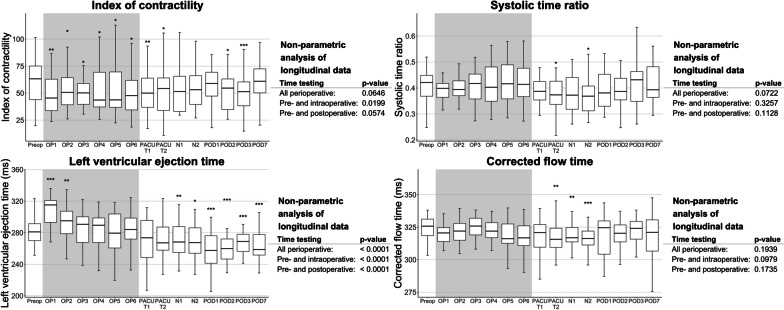
Perioperative time course of selected EC parameters (index of contractility, systolic time ratio, left ventricular ejection time, corrected flow time). Data are shown as boxplots over time from the preoperative baseline up to the postoperative day 7 (POD7). Asterisks indicate (*) *p* < 0.05, (**) *p* < 0.01, and (***) *p* < 0.001 of the respective timepoint versus the preoperative baseline values according to the non-parametric analysis for longitudinal data. The non-parametric analyses of longitudinal data for the parameters over the perioperative period are indicated on the right-hand side, respectively. *BL* baseline, *N1-2* timepoints 1–2 during the first postoperative night, *OP1-6* timepoints 1–6 in surgery, *PACU T1* postanaesthesia care unit timepoint 1, *PACU T2* postanaesthesia care unit timepoint 2, *POD1-3/7* postoperative days 1–3/7

Longitudinal analysis of perioperative haemodynamic data overall (Figs. 2,3) presented no differences in SVI, index of contractility, corrected flow time, and systolic time ratio over time. However, changes over time were detected for HR, which was reduced intraoperatively and elevated postoperatively, and CI, which was decreased especially intraoperatively. The thoracic fluid volume was elevated over the entire perioperative period. For the index of contractility, only the time course pre- and intraoperative showed significant changes with reduced contractility. Also, the left ventricular ejection time was significantly different, which can be explained by the change in HR.

**Fig. 4:**
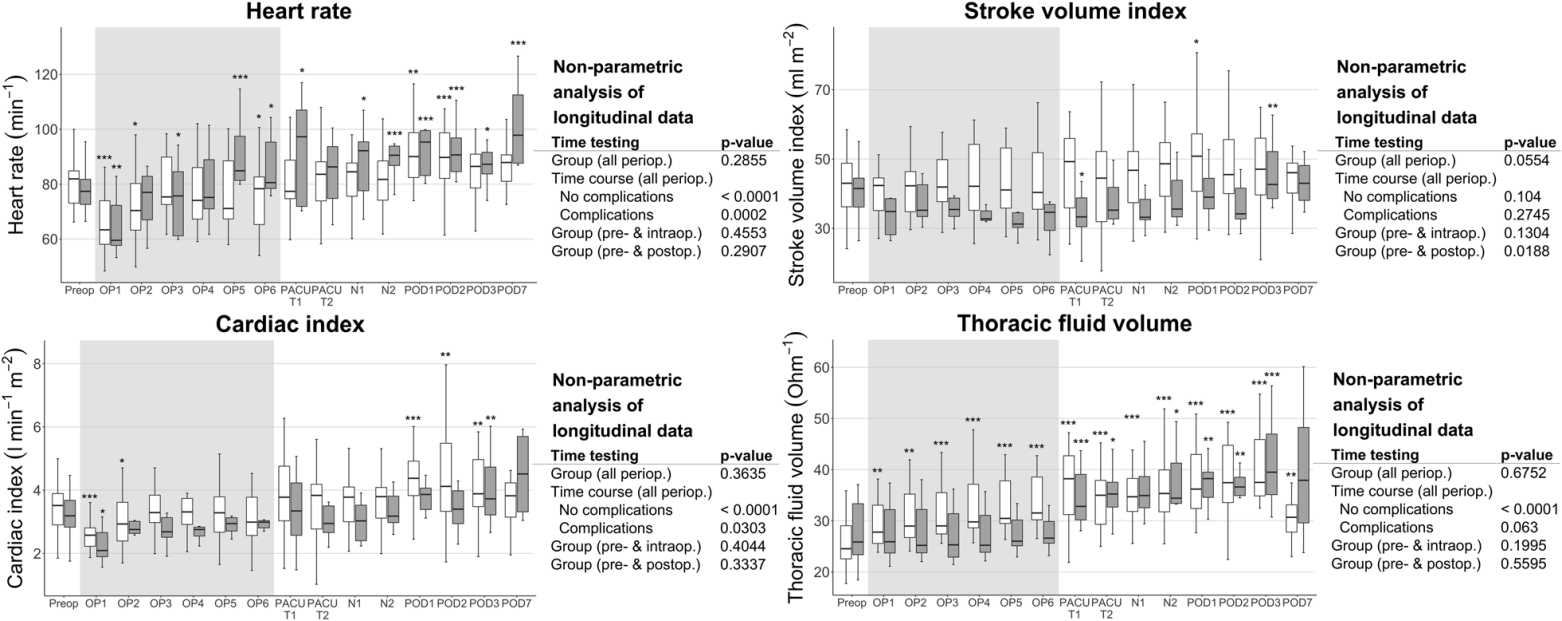
Perioperative longitudinal data of selected EC parameters (heart rate, stroke volume index, cardiac index, thoracic fluid content), grouped by the incidence of a complication according to Clavien–Dindo higher than IIIa (  no complications,  complications). Data are shown as boxplots over time from the preoperative baseline up to the postoperative day 7 (POD7). Asterisks indicate (*) *p* < 0.05, (**) *p* < 0.01, and (***) *p* < 0.001 of the respective timepoint versus the preoperative baseline values according to the non-parametric analysis for longitudinal data. The non-parametric analyses of longitudinal data for the parameters over the perioperative period are indicated on the right-hand side, respectively. *BL* baseline, *N1-2* timepoints 1–2 during the first postoperative night, *OP1-6* timepoints 1–6 in surgery, *PACU T1* postanaesthesia care unit timepoint 1, *PACU T2* postanaesthesia care unit timepoint 2, *POD1-3/7* postoperative days 1–3/7

**Fig. 5:**
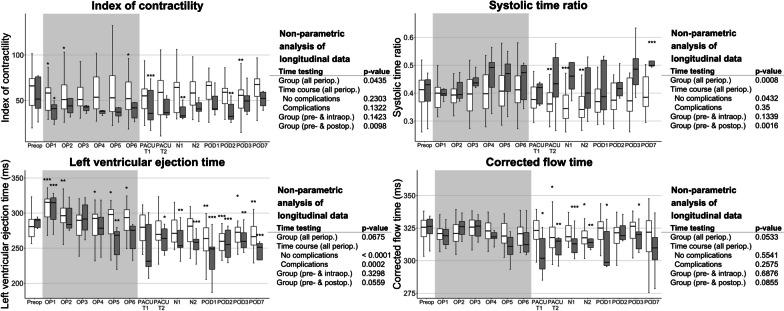
Perioperative longitudinal data of selected EC parameters (index of contractility, systolic time ratio, left ventricular ejection time, corrected flow time), grouped by the incidence of a complication according to Clavien–Dindo higher than IIIa (  no complications,  complications). Data are shown as boxplots over time from the preoperative baseline up to the postoperative day 7 (POD7). Asterisks indicate (*) *p* < 0.05, (**) *p* < 0.01, and (***) *p* < 0.001 of the respective timepoint versus the preoperative baseline values according to the non-parametric analysis for longitudinal data. The non-parametric analyses of longitudinal data for the parameters over the perioperative period are indicated on the right-hand side, respectively. *BL* baseline, *N1-2* timepoints 1–2 during the first postoperative night, *OP1-6* timepoints 1–6 in surgery, *PACU T1* postanaesthesia care unit timepoint 1, *PACU T2* postanaesthesia care unit timepoint 2, *POD1-3/7* postoperative days 1–3/7

A comparison of groups according to postoperative complications over the perioperative time course (Figs. 4 and 5) revealed significant differences between patients with and without complications for the parameters index of contractility and systolic time ratio. For only the pre- to post-operative time course, also SVI showed significant differences between groups. Patients with postoperative complications had a lower SVI and index of contractility postoperatively and a prolonged systolic time ratio perioperatively. Analogous to the changes over time detected overall, HR and CI showed changes over time for each group (no complications versus complications) separately. Regarding the systolic time ratio and the corrected flow time, only the group without complications changed over time.

In the longitudinal analysis of the inflammatory markers interleukin-6 (IL-6) and intercellular adhesion molecule 1 (ICAM-1) (Additional file 3: Figure S1 and Additional file 4: Figure S2), we found changes over time for the entire population and each group separately. Both markers showed an increase after surgery. The only exception was the longitudinal analysis of ICAM-1 in patients without complications, which did not show any changes over time. Furthermore, both markers detected systematic differences between groups with higher values for patients with complications. Maximum values were reached at timepoint T1 for IL-6 and POD1 for ICAM-1.

Baseline characteristics showed no significant differences between the groups with and without complications, except for a higher age and a counterintuitively higher Charlson Comorbidity Index in patients without complications but with a low overall comorbidity (Table 1). Preoperatively, all patients featured a low individual cardiovascular risk and were rather healthy, apart from their cancer diagnosis. In all patients, an arterial and central venous line, as well as an epidural catheter was placed. After the induction of anaesthesia with propofol and remifentanil or fentanyl, the anaesthesia was maintained by sevo- or desflurane, remifentanil and or fentanyl, and the epidural administration of ropivacaine 0.2% and sufentanil.

**Table 1:** Descriptive preoperative data of patient characteristics

	Overall	No complications	Complications	*p*-value
*n*	29	22	7	
Age (years)	59 [50, 61]	56 [50, 60]	64 [59, 68]	0.049
BMI (kg m^−2^)	24.2 [21.8, 27.9]	24.9 [21.8, 27.7]	23.3 [22.1, 26.6]	0.823
ASA classification				
1	3 (10)	1 (5)	2 (29)	0.221
2	17 (59)	14 (64)	3 (43)	
3	9 (31)	7 (32)	2 (29)	
NYHA classification				
1	23 (79)	18 (82)	5 (71)	0.612
2	6 (21)	4 (18)	2 (29)	
Apfel score (PONV)				
1	0 (0)	0 (0)	0 (0)	1.000
2	3 (10)	2 (9)	1 (14)	
3	18 (62)	14 (64)	4 (57)	
4	8 (28)	6 (27)	2 (29)	
MET score	5 [3, 6]	5 [3, 6]	5 [3, 6]	0.917
Comorbidities und medication				
Charlson Comorbidity Index	2 [2, 3]	3 [2, 6]	2 [2]	0.017
Arterial hypertension	8 (28)	6 (27)	2 (29)	1.000
Antihypertensive medication	8 (28)	6 (27)	2 (29)	1.000
Other long-term medication	29 (100)	22 (100)	7 (100)	1.000
POSSUM Score				
Morbidity	53.5 [38.5, 62.5]	53.4 [33.6, 62.5]	55.0 [42.8, 66.5]	0.524
Mortality	2.6 [1.7, 3.9]	2.6 [1.5, 3.8]	3.3 [2.0, 4.2]	0.523

Data are shown as median [Q25, Q75] or as n-number (%). *p*-values were calculated with the Wilcoxon–Mann–Whitney rank-sum test for metric data and the Fisher’s exact test for categorical variables. *ASA* American Society of Anaesthesiologists, *BMI* body mass index, *MET score* metabolic equivalent of task activity-score, *NYHA* New York Heart Association, *PONV* postoperative nausea and vomiting, *POSSUM Score* physiological and operative severity score for the enumeration of mortality and morbidity

During surgery, patients with postoperative complications required a higher maximum rate of norepinephrine than patients without complications, whereas the cumulative dose needed was comparable. Also, their intraoperative urine output was significantly lower. There were no differences in intraoperative fluid management and blood loss, duration of surgery or anaesthesia, and the length of stay in the postanaesthesia care unit (PACU) or ICU (Table 2).

**Table 2:** Descriptive perioperative data

	Overall	No complications	Complications	*p*-value
*n*	29	22	7	
Norepinephrine, highest rate (µg kg^−1^ min^−1^)	0.10 [0.10, 0.20]	0.10 [0.09, 0.14]	0.28 [0.17, 0.30]	0.003
Norepinephrine, cumulative (µg)	1530 [1001, 1922]	1466 [863, 1736]	1922 [1296, 2957]	0.149
Crystalloids (ml)	3000 [2500, 3500]	3000 [2500, 3000]	3000 [2750, 3500]	0.360
Colloids (ml)	1000 [1000, 1000]	1000 [1000, 1000]	1000 [1000, 1250]	0.374
FFP (IE)	1320 [660, 2420]	1320 [660, 2145]	2248 [1320, 2530]	0.124
RBC concentrate (IE)	0 [0, 310]	0 [0, 0]	310 [0, 930]	0.076
Urine output (ml)	660 [275, 800]	690 [470, 1110]	275 [220, 425]	0.036
Blood loss (ml)	500 [300, 1000]	500 [300, 938]	800 [700, 2000]	0.091
Duration of surgery (h)	4.4 [4.0, 5.0]	4.7 [4.0, 5.1]	4.1 [3.6, 4.6]	0.251
Duration of anaesthesia (h)	6.1 [5.8, 6.8]	6.2 [5.8, 6.8]	6.0 [5.6, 6.1]	0.372
Length of stay in PACU or ICU (h)	22.0 [19.6, 46.6]	21.9 [7.7, 38.8]	43.0 [21.5, 122.4]	0.217
Length of stay in hospital (d)	11.7 [11.5, 17.6]	11.6 [10.7, 14.4]	24.7 [18.1, 45.2]	0.002

Data are shown as median [Q25, Q75]. *p*-values were calculated with the Wilcoxon–Mann–Whitney rank-sum test. *FFP* fresh frozen plasma, *ICU* intensive care unit, *PACU* postanaesthesia care unit; RBC, red blood cell

The median SOFA score on the first postoperative day was four points (corresponding to a mortality of 20%) and did not differ between groups. On the seventh postoperative day the SOFA score was significantly higher for patients with complications (with a median of two points) in comparison to patients without complications. Furthermore, the patients showed significant differences in the APACHE-II at both time points (postoperative day 1/7) with a higher mortality in the complication group (12% mortality during hospitalisation).

Data concerning cancer surgery and postoperative tumour staging were evaluated. Both groups presented a quite advanced tumour staging and grading, without significant differences. Regarding tumour expansion, patients with complications more often showed a more extensive tumour spread. Consequently, the surgical procedures of colectomy, colostomy, and splenectomy were performed more often.

## Discussion

In this study, we have shown with EC that patients undergoing extended cancer surgery due to ovarian cancer show substantial haemodynamic changes throughout the perioperative course. From the preoperative to the first intraoperative timepoint, we measured relatively decreased values for HR, CI, SVI, and the index of contractility. HR and CI were decreased intraoperatively and increased postoperatively indicating a context-sensitive pattern, whereas the index of contractility was decreased only intraoperatively. Thoracic fluid volume increased continuously throughout surgery and the postoperative phase and did not normalise up to hospital discharge. Patients having postoperative complications differed from those without complications in a lower SVI and index of contractility postoperatively and a prolonged systolic time ratio perioperatively, IL-6 and ICAM-1 were both elevated postoperatively.

Our data indicate that after induction of anaesthesia, the patients had a marked reduction in sympathetic tone with a reduced HR and cardiac inotropic state indicated by the index of contractility. Consequently, there was a reduced SVI despite longer diastolic filling times and a substantially decreased CI. In contrast, we saw no change in the corrected flow time as a marker of afterload, indicating that the vasodilatory effects of the anaesthetic drugs and the epidural anaesthesia were counteracted in our study by the continuous norepinephrine administration. As norepinephrine is acting on the venous capacitance vessels, we can hypothesise that venous pooling might have been partially counteracted. Whereas we cannot definitively exclude central hypovolaemia as the reason for a decreased SVI, a decreased preload can reduce inotropy, but cannot explain the reduced HR. Consequently, we consider the observed haemodynamic changes primarily as an expression of a reduced sympathetic tone.

In patients undergoing hip arthroplasty within a pathway for enhanced recovery after surgery, early postoperative mobilisation was impaired by orthostatic hypotension and intolerance due to an attenuated sympathetic activity, a relatively elevated parasympathetic response, or a delay in vascular responsiveness [29]. A perioperative autonomic disbalance due to a reduced sympathetic reactivity could also explain our intraoperative data. This disbalance might be caused by excessive measures applied to reduce perioperative stress like opioids or regional anaesthesia. Nevertheless, a peridural catheter reduces postoperative complications and long-term mortality [30]. The guided administration of catecholamines to counteract autonomic disbalance seems to be a rational approach. Yet, our data do not suggest the general administration of positive inotropic drugs, it could be individualised according to the changes of advanced haemodynamic parameters like the index of contractility in relation to the patient’s baseline and weaned out throughout surgery if it normalises. This approach could be like haemodynamic optimisation pathways that propose the use of positive inotropic agents to avoid a CI decrease [31, 32].

Another study performed non-invasive preoperative haemodynamic measurements as a baseline for an intraoperative individualised, goal-directed algorithm to guide fluid and catecholamine administration [3]. In 57% of patients, the first intraoperative CI value was below the preoperative value, which is compatible with our observation: in comparison to the preoperative baseline, HR and CI were decreased throughout the surgery. Over time, a continuous increase was recorded up to the first to third postoperative day. In contrast, IL-6 peaked one hour after surgery, expressing systemic inflammation due to surgical trauma, and was already decreasing on the following postoperative days. Because of these differences in progression, we suggest that on the day of surgery, the decreased sympathetic tone under anaesthesia masks the hyperdynamic state resulting from systemic inflammation.

The SVI recovered from its reduction on the first intraoperative timepoint and then stabilised at preoperative values, whereas the index of contractility remained reduced throughout the surgery. As the thoracic fluid volume increased continuously from the start of surgery up to the third postoperative day, the data suggest that the SVI was maintained by fluid administration despite the negative inotropic effects of the anaesthesia. However, due to the continuously increasing thoracic fluid volume, we cannot exclude that the data were an expression of intraoperative fluid overload.

Noblett and colleagues showed differences in CI between a conventional and an intervention group where fluid administration was guided by oesophageal Doppler measurements [33]. This was achieved by individually timed fluid administration, while the overall administration of crystalloids or colloids during surgery did not differ between groups, which showed that timing and individualisation of fluid administration are relevant. Accordingly, based on the index of contractility values of our data, one could raise the hypothesis that an individually guided administration of inotropic drugs could be beneficial to maintain cardiocirculatory flow and reduce excess fluid administration. In contrast to established goal-directed therapy algorithms with invasive monitoring, EC could be a method to guide goal-directed therapy in a broader patient population due to its non-invasive nature.

Comparing patients with and without postoperative complications, we saw postoperatively decreased SVI and index of contractility values in patients with complications. The corrected flow time as a marker of intravascular volume was reduced on various postoperative timepoints versus the baseline in patients with complications. Together with the increased perioperative values of ICAM-1 our data suggest a reduction of intravascular circulating volume due to increased extravasation of fluids contributing to the decreased SVI.

The systolic time ratio is a sensitive cardiovascular marker, an increased ratio correlates with a reduced left ventricular ejection fraction [17, 18]. It has been shown that decreases of SVI by upright positioning or forced diuresis cause a decrease in SVI and are associated with an increased systolic time ratio [19, 20]. Between patients with and without complications, the systolic time ratio was elevated perioperatively and especially postoperatively in patients with complications, supporting the point that it could be a possible marker to determine patients at risk for relevant postoperative complications.

It should be emphasised that the present study was merely designed in an exploratory fashion and thus does not allow for generalisation or evidence. Moreover, our observations were based on a small and only female population. Further, prospective, and interventional studies are required to explore if these findings are reproducible in other surgeries with a similar medium to high risk profile and to show whether preoperatively started treatment algorithms based on CO monitoring can reduce complications.

The reliability of the measured values cannot be proven, because we did not employ a second CO measurement method. However, in a longitudinal data analysis, trending characteristics are more important than absolute values and proving the reliability of the method was not the goal of this study. This is compatible with the meta-analysis of Sanders and colleagues indicating that EC could complement monitoring, providing a continuous monitoring [7].

## Conclusion

In summary, the data show substantial changes of haemodynamic parameters (HR, SVI, CI, and index of contractility) from the day prior to surgery to the first measurement after induction of anaesthesia and the start of surgery. Longitudinally over the perioperative time course, the CI was relevantly decreased during surgery and increased over the postoperative period.

Patients with postoperative complications differed from patients without complications in the markers of cardiac function. This indicates that deviating postoperative haemodynamic parameters might be associated with the development of postoperative complications.

Thus, the analyses of trends over the whole perioperative time course by using non-invasive technologies like EC seem to be useful to identify patients with altered haemodynamic parameters and therefore at an increased risk for postoperative complications after major surgery with a moderate or high perioperative risk.

### Supplementary Information

**Additional file 1: Text S1.** Text with explanation of haemodynamic parameters.

**Additional file 2: Table S1.** Table of haemodynamic parameters.

**Additional file 3: Figure S1.** Perioperative Longitudinal Data of IL-6 and ICAM-1. Data are shown as boxplots over time from the preoperative baseline up to the postoperative day 7 (POD7). Asterisks indicate (*) *p* < 0.05, (**) *p* < 0.01, and (***) *p* < 0.001 of the respective timepoint versus the preoperative baseline values according to the non-parametric analysis for longitudinal data. The non-parametric analyses of longitudinal data for the parameters over the perioperative period are indicated on the right-hand side, respectively. Abbreviations: ICAM-1, intercellular adhesion molecule-1; IL-6, interleukin-6.

**Additional file 4: Figure S2.** Perioperative Longitudinal Data of IL-6 and ICAM-1, Grouped by the Incidence of a Complication According to Clavien–Dindo Higher Than IIIa (Non shaded—No Complications, Shaded—Complications). Data are shown as boxplots over time from the preoperative baseline up to the postoperative day 7 (POD7). Asterisks indicate (*) *p* < 0.05, (**) *p* < 0.01, and (***) *p* < 0.001 of the respective timepoint versus the preoperative baseline values according to the non-parametric analysis for longitudinal data. The non-parametric analyses of longitudinal data for the parameters over the perioperative period are indicated on the right-hand side, respectively. Abbreviations: ICAM-1, intercellular adhesion molecule-1; IL-6, interleukin-6.

## Data Availability

The datasets used and/or analysed during the current study are available from the corresponding author upon reasonable request.

## Abbreviations

ASA: American Society of Anaesthesiologists
APACHE II Score: Acute Physiology and Chronic Health Evaluation II Score
BL: Baseline
BMI: Body mass index
CO: Cardiac output
CI: Cardiac index
EC: Electrical cardiometry
FFP: Fresh frozen plasma
HR: Heart rate
ICAM-1: Intercellular adhesion molecule-1
ICON: Index of contractility
IL-6: Interleukin-6
ICU: Intensive care unit
MET: Metabolic equivalent of task
N1-2: Timepoints 1–2 during the first postoperative night
NYHA: New York Heart Association
OP1-6: Timepoints 1–6 in surgery
PACU: Postanaesthesia care unit
PACU T1: Postanaesthesia care unit timepoint 1
PACU T2: Postanaesthesia care unit timepoint 2
POD1-3/7: Postoperative days 1–3/7
PONV: Postoperative nausea and vomiting
POSSUM Score: Physiological and operative severity score for the enumeration of mortality and morbidity
RBC: Red blood cell
SOFA Score: Sequential Organ Failure Assessment
STR: Systolic time ratio
SVI: Stroke volume index
