# Supplementary material for: Perioperative advanced haemodynamic monitoring of patients undergoing multivisceral debulking surgery: an observational pilot study
Source: Intensive Care Med Exp. 2023 Sep 8;11:61. doi: 10.1186/s40635-023-00543-1 (PMC10491568; doi:10.1186/s40635-023-00543-1)
Supplement: Supplementary file 1 — Additional file 1: Text S1. Text with explanation of haemodynamic parameters. [file 40635_2023_543_MOESM1_ESM.docx]

**SUPPLEMENTAL FILE 1: Text S1. Haemodynamic parameters**

Beside the common haemodynamic parameters, such as HR, SVI, and CI, the algorithm of electrical cardiometry also measures the parameters systolic time ratio (STR), the thoracic fluid volume (TFC) and the index of contractility (ICON). The index of contractility correlates positively with the myocardial contraction strength [13, 14], and the thoracic fluid volume is a marker for the total thoracic fluid volume (intra- and extravascular) [15, 16]. The systolic time ratio is calculated from the pre-ejection period (electrical systole) and the left ventricular ejection time (LVET, mechanical systole). Together they sum up to the systolic interval of the cardiac cycle. The pre-ejection period is prolonged when myocardial contractility is impaired due to the decreased ability of the myocardium to increase the intracardiac pressure prior to the ejection period [17, 18]. Another reason for a prolonged pre-ejection period is hypovolemia as the decreased preload exerts less contraction potentiation, as stated by the Frank-Starling mechanism [19, 20]. The left ventricular ejection time is reduced with increased afterload and consequently reduced preload states.^21^ The systolic time ratio is, therefore, a sensitive cardiovascular marker and an increased ratio correlates, according to numerous cardiac studies, well with the reduced left ventricular ejection fraction [17, 18]. It has further been shown that decreases of SVI by upright positioning or forced diuresis cause a decrease in SVI and are associated with an increased systolic time ratio [19, 20]. Consequently, the systolic time ratio is a sensitive parameter to changes in both pre- and afterload. The left ventricular ejection time is inversely proportional to the HR. Therefore, the left ventricular ejection time is standardised to the HR, and then called corrected flow time (FTc) [17, 22].

Abbreviations: CI, cardiac index; HR, heart rate; SVI, stroke volume index.
