# Supplementary material for: Perioperative advanced haemodynamic monitoring of patients undergoing multivisceral debulking surgery: an observational pilot study
Source: Intensive Care Med Exp. 2023 Sep 8;11:61. doi: 10.1186/s40635-023-00543-1 (PMC10491568; doi:10.1186/s40635-023-00543-1)
Supplement: Supplementary file 2 — Additional file 2: Table S1. Table of haemodynamic parameters. [file 40635_2023_543_MOESM2_ESM.docx]

**Table S1: Table of haemodynamic parameters** (adapted from Stetzuhn et al [23])

| **Abbreviation** | **Name of parameter** | **Unit** | **Definition** |
| --- | --- | --- | --- |
| SV | Stroke volume | ml | Ejected blood volume from the left ventricle during systole |
| HR | Heart rate | 1·min^-1^ | Cardiac cycles / minute |
| CO | Cardiac output | l·min^-1^ | SV / HR |
| SVI | Stroke volume index | ml·m^-2^ | SV / body surface area |
| CI | Cardiac index | l·min^-1^·m^-2^ | CO / body surface area |
| PEP | Pre-ejection period | ms | Time from beginning of the chamber complex measured by ECG to the ejection of blood from the left ventricle |
| LVET | Left ventricular ejection time | ms | Duration of systolic blood ejection |
| FTC | Corrected flow time | ms | Frequency corrected LVET using Bazett´s formula |
| STR | Systolic time ratio |  | PEP / LVET |
| ICON | Index of contractility |  | Peak acceleration of erythrocytes in the aorta, calculation described in ^24^ |
| TFC | Thoracic fluid volume | 1·Ohm^-1^ | Total thoracic fluid volume (intra- and extravascular) |
