# Supplementary figures and images for: Perioperative advanced haemodynamic monitoring of patients undergoing multivisceral debulking surgery: an observational pilot study
Source: Intensive Care Med Exp. 2023 Sep 8;11:61. doi: 10.1186/s40635-023-00543-1 (PMC10491568; doi:10.1186/s40635-023-00543-1)

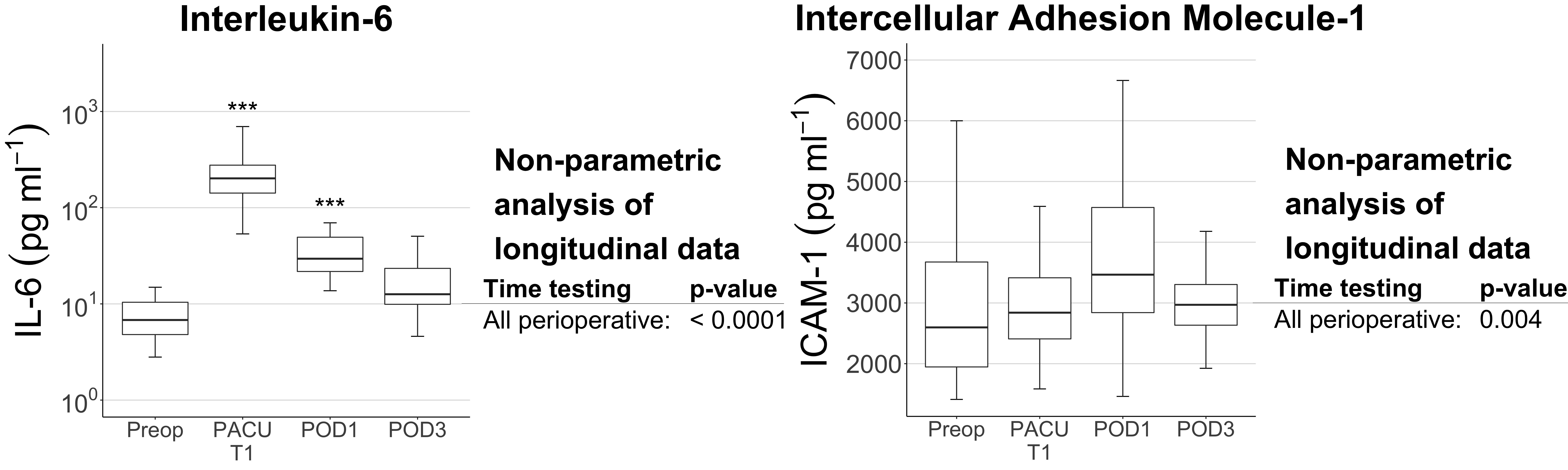

Supplement: Supplementary file 3 — Additional file 3: Figure S1. Perioperative Longitudinal Data of IL-6 and ICAM-1. Data are shown as boxplots over time from the preoperative baseline up to the postoperative day 7 (POD7). Asterisks indicate (*) p < 0.05, (**) p < 0.01, and (***) p < 0.001 of the respective timepoint versus the preoperative baseline values according to the non-parametric analysis for longitudinal data. The non-parametric analyses of longitudinal data for the parameters over the perioperative period are indicated on the right-hand side, respectively. Abbreviations: ICAM-1, intercellular adhesion molecule-1; IL-6, interleukin-6. [file 40635_2023_543_MOESM3_ESM.tiff]

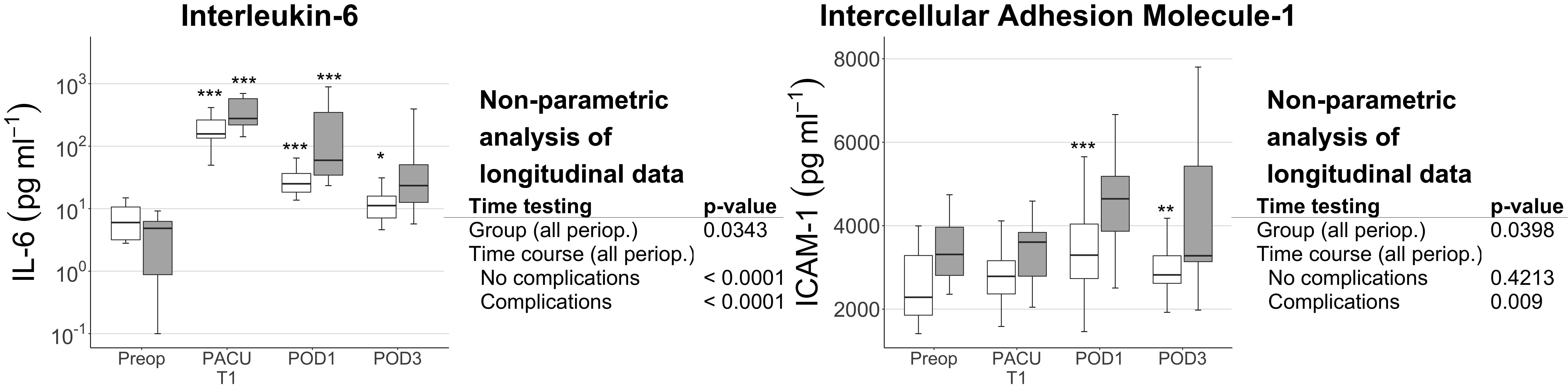

Supplement: Supplementary file 4 — Additional file 4: Figure S2. Perioperative Longitudinal Data of IL-6 and ICAM-1, Grouped by the Incidence of a Complication According to Clavien–Dindo Higher Than IIIa (Non shaded—No Complications, Shaded—Complications). Data are shown as boxplots over time from the preoperative baseline up to the postoperative day 7 (POD7). Asterisks indicate (*) p < 0.05, (**) p < 0.01, and (***) p < 0.001 of the respective timepoint versus the preoperative baseline values according to the non-parametric analysis for longitudinal data. The non-parametric analyses of longitudinal data for the parameters over the perioperative period are indicated on the right-hand side, respectively. Abbreviations: ICAM-1, intercellular adhesion molecule-1; IL-6, interleukin-6. [file 40635_2023_543_MOESM4_ESM.tiff]
